# A novel mutation in the SH3BP2 gene causes cherubism: case report

**DOI:** 10.1186/1471-2350-7-84

**Published:** 2006-12-05

**Authors:** Cui-Ying Li, Shi-Feng Yu

**Affiliations:** 1 Department of Oral Pathology, School of Stomatology, Peking University, Beijing, China

## Abstract

**Background:**

Cherubism is a rare hereditary multi-cystic disease of the jaws, characterized by its typical appearance in early childhood, and stabilization and remission after puberty. It is genetically transmitted in an autosomal dominant fashion and the gene coding for SH3-binding protein 2 (SH3BP2) may be involved.

**Case presentation:**

We investigated a family consisting of 21 members with 3 female affected individuals with cherubism from Northern China. Of these 21 family members, 17 were recruited for the genetic analysis. We conducted the direct sequence analysis of the SH3BP2 gene among these 17 family members. A disease-causing mutation was identified in exon 9 of the gene. It was an A1517G base change, which leads to a D419G amino acid substitution.

**Conclusion:**

To our knowledge, the A1517G mutation has not been reported previously in cherubism. This finding is novel.

## Background

Cherubism (MIM 118400) is an autosomal dominant inherited disease of the jaws. The lesion was first described by Jones in 1933 as a hereditary multilocular cystic disease of the jaws, characterized by symmetrically swollen cheeks, particularly over the angles of the mandible, and an upward turning of the eyes. The author coined the descriptive term cherubism in 1938 because the typical appearance resembled cherubism found in Renaissance art [1]. The affected mandible and maxilla begin to swell in early childhood, and are gradually increased until the age of puberty [2]. The WHO has classified cherubim as a non-neoplastic bone lesion. According to a latest literature search, there have been about 200 cases reported previously, including some sporadic cases [3].

The characteristic radiographic findings in cherubism are well-defined as being multilocular areas of diminished density, often very extensive, with a few irregular bony septa. In the adult, the multilocular rarefactions become replaced by sclerosis, with progressive calcification [4]. The dysplasia histologically consists of a mononuclear fibroblastic stroma with large numbers of multinucleated giant cells and cyst formation [5], but the characteristic perivascular collagen cuffing is sometimes present. At the periphery of the lesions, there may be newly formed osteoid and bones. Such a histological feature can be distinguished from other giant cell-rich lesions as it has been found only in the jaws of patients with cherubism. In addition to the typical histological features, therefore, the bilateral involvement of the jaws, the typical course, and the familial occurrence of the disease are required for the diagnosis of cherubism.

The gene responsible for cherubism has been mapped to the region of chromosome 4p16.3 [6,7]. Point mutations in the gene coding for SH3BP2 have been identified in 12 of 15 families. All the mutations identified so far are present in exon 9 of the gene and cause amino acid substitutions within a 6-amino acid sequence [8]. Several studies of cherubism have been reported in Chinese families, but the work is restricted to clinical description only [9-11]. In the present study, therefore, we attempted to perform the direct sequence analysis of the SH3BP2 gene in a family with multiple affected individuals with cherubism to see if there would be a novel mutation causing the illness in the Chinese family.

## Case presentation

### Subjects

**Figure 1:**
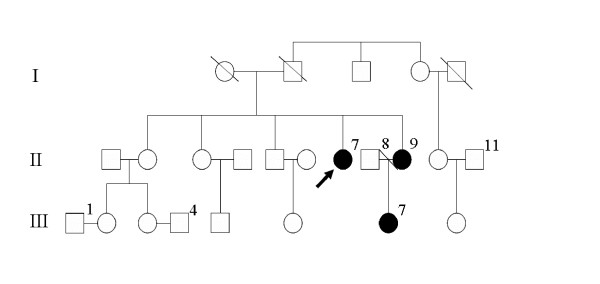
Pedigree of the family with three female affected individuals. The filled symbol represents the affected individuals and the arrow indicates the proband (member II-7).

**Figure 2:**
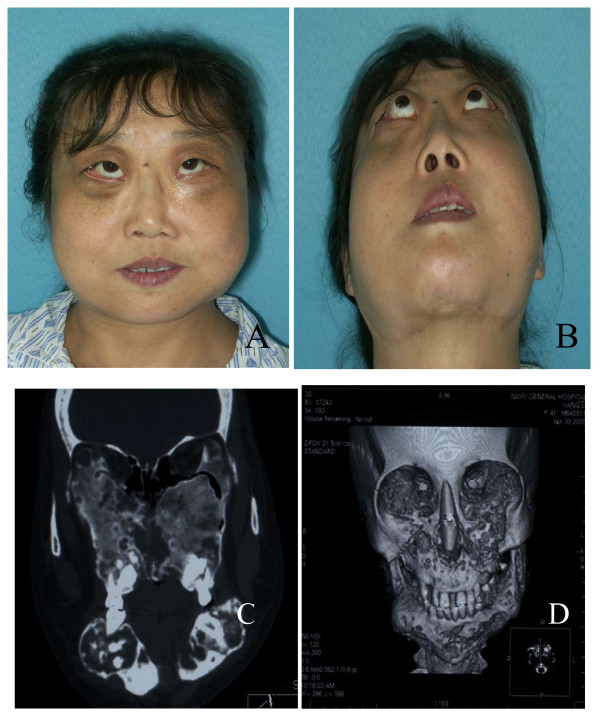
Facial appearances and panoramic radiophotographic findings of affected member II-7. The facial appearances show a symmetrical swelling of the cheeks and an exophthalmos (A) as well as the mandibular angles (B). Computed tomography scans show bilateral lesional tissue expanding the anteriorwall of the maxilla and the body of the mandible (C). Three-dimensional computed tomography shows a remarkable expansion of the mandible, which can lead to a soap bubble appearance and the bilateral bulges of the maxilla (D).

A family consisting of 21 biologically related members, 3 of whom were female patients with cherubism, was identified in Northern China. Seventeen members were recruited for the present study as four male members II-8, II-1, III-1 and III-4 were missing. As shown in Figure 1, affected member II-7 was the proband of cherubism, aged 41 years, with a slowly progressive upward displacement of both eyes and an intraorbital mass bilaterally (Figure 2A). An irregular mandible was noticed at the age of 10 years. Her facial appearance was typically cherubic. Radiological examinations demonstrated bilateral multi-cystic lesions both in mandible and in maxilla compatible with cherubism. She was promptly referred to a maxillofacial service. An incisional biopsy was performed. Histological examination revealed many giant cells in a vascularized fibrous stroma. The diagnosis of cherubism was confirmed, and surgical operations, such as curettage and osteoplasty, were then performed. Although cherubism is generally self-limiting and subsides with age, the patient described here was unusual in that the orbital involvement arose in adulthood after the jaw lesions had subsided. A physical examination revealed symmetrical enlargement of the jaws, exophthalmos with diplopia and a slight upward turning of the eyes (Figure 2A). The mandibular angles were also observed (Figure 2B). Her visual activity and field were impaired. Computed tomography (CT) scans showed bilateral multi-cystic lesions in the maxilla and mandible (Figure 2C). The CT scans also revealed that the lesions consisted mainly of soft tissue masses with a ballooned cortex. Furthermore, the lesion of the both maxilla extended into the orbit and then affected the eyeball position (Figure 2C). However, there was no sign of compression of the optic canal by the lesion. Three-dimensional CT scans revealed that the symmetrically expanded anterior walls of the maxilla and the symmetrically expanded mandible had a soap bubble appearance as described previously by James and co-workers [2] (Figure 2D).

**Figure 3:**
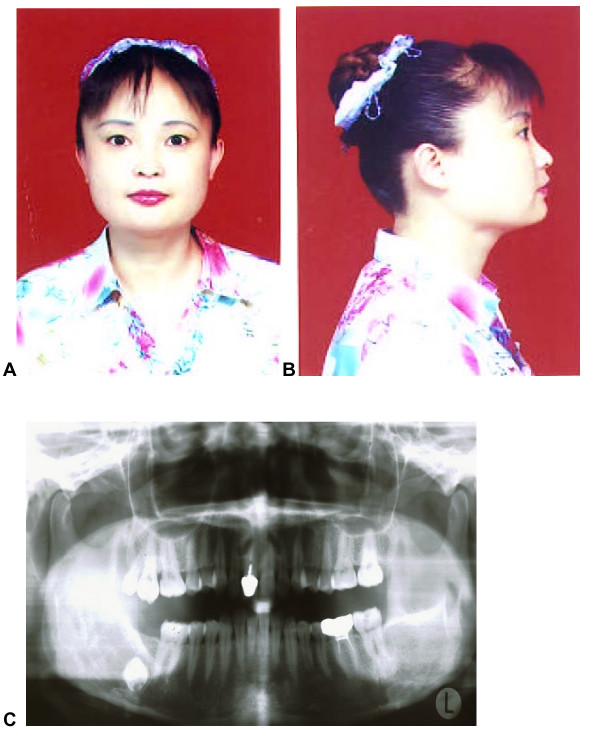
Facial appearances and panoramic radiophotographic findings of affected member II-9.

The affected member II-9 is a sister of the member II-7, aged 37 years. She was diagnosed as having cherubism at the age of 7 years and she had fibrous dysplastic tissue curetted from her bilateral mandibles. A pathological examination confirmed clusters of multinucleated giant cells within a fibrous stroma, consistent with the diagnosis of cherubism. Because she had undergone a surgery operation, her facial appearances looked well when her sample was taken for this study (Figure 3A and 3B). However, the jaw panoramic radiophotography showed partial calcifications of anterior mandible and mandibular angles (Figure 3C).

**Figure 4:**
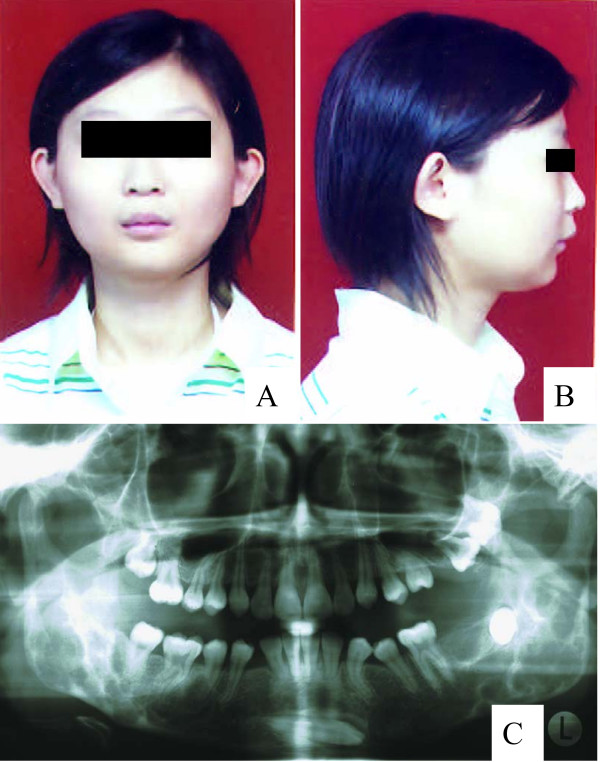
Facial appearances and panoramic radiophotographic findings of affected member III-7.

The affected member III-7 is the daughter of the member II-9, aged 14 years. She developed bilateral cystic lesions of the mandible when she was 8 or 9 years old. At the age of 9 years, there were visible lesions over the bilateral mandibular angles and radiographs showed symmetric multi-cystic lesions. Osteoplasty of the affected jaws was performed. Her facial appearances did not show severe abnormalities (Figure 4A and 4B) although the jaw panoramic radiophotography revealed symmetric multi-cystic alterations and impacted right mandibular canine teeth. Defect of left mandibular canine, second molar teeth and tooth root was also observed (Figure 4C).

To identify a disease-causing mutation, we also recruited 100 unrelated healthy subjects as control for DNA analysis.

All the subjects gave written informed consent for the present study, including clinical examinations, blood collection and genetic analysis. Written consent was also obtained from the patients and their relatives for publication of study. This study was approved by the local institutional review boards.

### Sequencing of the SH3BP2 gene

Genomic DNA for the genetic analysis was extracted from peripheral venous blood samples by conventional methods. The primers used for PCR amplification of exon 9 of the gene were designed based on its DNA sequence (accession numbers NT003023 and NM006081), including 5'-TgA gCT TTT TAg ggT CAC Agg-3'and 5'-ggC TTT ACA Tgg TgC TgT gT-3'. The PCR amplification was performed in a 25-μL reaction volume containing 100 ng of genomic DNA, 2.5 μL of 10X PCRx amplification buffer, 2.5 μL of 2.5 mM of dNTP mixture, 10 μM of each primer, and 1.0 U of Taq DNA polymerase. The conditions used for the PCR amplification included denaturation at 94°C for 30 seconds, followed by 40 cycles at 94°C for 30 seconds, 62°C for 30 seconds and 72°C for 30 seconds, and final elongation at 72°C for 8 minutes. The purified PCR products were used as templates for direct sequencing by a fluorescent dye-terminator cycle sequencing method with an ABI PRISM Big Dye terminator cycle sequencing ready reaction kit (Applied Biosystems, USA). Nucleotide sequences were determined by an ABI 3730 XL automated DNA sequencer (Applied Biosystems, USA).

### Identification of mutations

Genomic DNA sequencing was performed in all 17 subjects recruited in this study. A missense mutation was identified in exon 9 of the SH3BP2 gene in patients with cherubism. It was an A1517G base change, which leads to the D419G amino acid substitution. Of 17 family members, the A1517G base change was shown only in 3 affected individuals (affected members II-7, II-9 and III-7). We did not observe such a mutation in 100 unrelated controls. It is very likely that the A1517G base change is a disease-causing mutation.

## Conclusion

In this study, we identified a missense mutation at the SH3BP2 gene in a Chinese family with multiple affected individuals with cherubism. To our knowledge, this mutation has not been reported previously. This is a novel finding.

Since disease-causing mutations identified so far are all located in exon 9 of the gene, which can result in amino acid substitutions within a six-amino acid sequence, it can be concluded that exon 9 of the SH3BP2 gene is a mutation hotspot for cherubism. However, apart from the dominant cases, in which the mutation of the SH3BP2 gene may be the only genetic cause for cherubism, the disease sometimes occurs sporadically. A recent study has failed to identify a mutation within the SH3BP2 gene in the case of a 14-year-old boy with grade I cherubism diagnosed late [12]. Possibly, the other candidate genes may also be involved and the recessive pattern of inheritance cannot be ruled out in some cases.

Moreover, the present work demonstrates that the A1517G base change is shown only in three female patients whereas expressivity of the disease varies based on their facial appearances and panoramic radiophotographic findings (Figures 2, 3 and 4). All normal subjects and healthy relatives have failed to show such a point mutation. This finding seems to be inconsistent to the hypothesis of incomplete penetrance in females although Anderson and McClendon (1962) have reported that penetrance of cherubism is 100% in males and 50–70% in females [13]. Possibly, the mutation site affects the penetrance in females although abnormal inactivation of the X chromosome cannot be ruled out.

## Abbreviations

DNA: Deoxyribonucleic acid

PCR: Polymerase chain reaction

SH3BP2: SH3-binding protein 2

WHO: World Health Organization

## Competing interests

The author(s) declare that they have no competing interests.

## Authors' contributions

CL recruited all the subjects investigated, carried out the molecular genetic studies and drafted the manuscript. SY conceived of the study, participated in its design and coordination. Both authors read and approved the final manuscript.

## Pre-publication history

The pre-publication history for this paper can be accessed here:


